# The mediating effect of BMI in serum vitamin D related sleep deprivation based on the NHANES database

**DOI:** 10.3389/fnut.2025.1571985

**Published:** 2025-06-10

**Authors:** Boya Gong, Bei Xu, Chaoban Wang, Xia Guo

**Affiliations:** ^1^Department of Pediatrics, West China Second University Hospital, Sichuan University, Chengdu, China; ^2^Key Laboratory of Birth Defects and Related Diseases of Women and Children, Sichuan University, Ministry of Education, Chengdu, China; ^3^Department of Pediatrics, Baoding No. 1 Central Hospital, Baoding, China

**Keywords:** vitamin D, sleep deprivation, BMI, obesity, NHANES

## Abstract

**Background:**

Sleep deprivation are a widespread condition globally, posing significant risks to individuals’ physical and mental health. Existing studies have explored the associations between sleep deprivation, vitamin D, and obesity. However, there is limited research on the combined effects of serum vitamin D and obesity. To address this gap, we conducted a cross-sectional study using the NHANES database to investigate the mediating role of BMI in serum vitamin D-related sleep deprivation among Americans.

**Methods:**

Our study included 20,865 participants from the NHANES database (2007–2018, 2021–2023). Logistic regression was utilized to assess the relationship between vitamin D levels and sleep deprivation. Mediation analysis was performed to examine the role of obesity in the association between vitamin D and sleep deprivation.

**Results:**

Increased vitamin D levels coupled with a BMI below the 75th percentile significantly reduced the risk of sleep deprivation to 0.70 times (95% CI: 0.64–0.77; *p* < 0.001) that of individuals with insufficient vitamin D and a BMI above the 75th percentile (>33.1 kg/m^2^). Serum vitamin D showed a direct significant effect on reducing sleep deprivation risk (*β* total = 1.92*10^−3^, *P* dir < 0.001), and its indirect effect through BMI was also highly significant (*β* indir = 7.59*10^−5^, *P* indir < 0.001). In the fully adjusted model, compared with the VD Inadequate and BMI > 75% group, the VD Sufficient and BMI < 75% group had a reduced risk of sleep deprivation (OR = 0.7, 95% CI = 0.64–0.77).

**Conclusion:**

This study demonstrates that in American adults, vitamin D can influence sleep deprivation both directly and indirectly through its impact on BMI. Therefore, for individuals suffering from sleep deprivation, vitamin D supplementation might offer potential benefits. Enhancing vitamin D levels could mitigate sleep deprivation risks, particularly when associated with lower BMI values.

## Introduction

Sleep is a periodic, temporary functional state primarily regulated by neurobiological mechanisms. The National Sleep Foundation (NSF) recommends that young adults should aim for 7–9 h of sleep per night, while older adults should strive for 7–8 h (1–3). Despite these recommendations, 42.8% of American adults do not achieve the recommended sleep duration. Epidemiological studies have linked insufficient sleep to various health issues, including cancer, cardiovascular events, depression, cognitive decline, and increased mortality, highlighting significant public health concerns (2, 4, 5).

Some lipid - soluble vitamins ingested are considered to be involved in various physiological regulatory functions, such as bone metabolism, metabolic syndrome, and obesity (6–8). Vitamin D, a fat-soluble vitamin and prohormone, is produced in the skin when ultraviolet B (UVB) radiation from sunlight interacts with 7-dehydrocholesterol. (9) Levels of 25-hydroxyvitamin D (25(OH)D) below 20 ng/mL (50 nmol/L) indicate deficiency, while levels between 21–29 ng/mL (52–72 nmol/L) suggest insufficiency. Levels of 30 ng/mL (75 nmol/L) or higher are considered adequate. It is estimated that approximately one billion people worldwide may suffer from vitamin D deficiency or insufficiency (10). Vitamin D is crucial for maintaining bone health and has been shown to play a role in reducing the risk of various diseases, including cardiovascular disease, cancer, autoimmune disorders, and infections (10, 11). Previous research indicates that vitamin D affects sleep through the presence of 1α-hydroxylase and 1,25-dihydroxyvitamin D receptors (VDR) in the brain. Studies show that 1,25-dihydroxyvitamin D can regulate the expression of circadian clock genes such as BMAL1 and PER2 in adipose-derived stem cells (12, 13). VDR is highly expressed in brain regions like the anterior and posterior hypothalamus, substantia nigra, periaqueductal gray matter, raphe nuclei, and pontine and caudal reticular nuclei, which are essential for regulating the sleep–wake cycle and muscle atonia during sleep (12). When vitamin D binds to these receptors, it influences sleep patterns and quality. Additionally, vitamin D may affect sleep quality by regulating tryptophan hydroxylase (TPH)-2, an enzyme responsible for converting tryptophan to 5-hydroxytryptophan. Fiammetta Romano et al. reported a positive correlation between vitamin D levels and sleep duration in American adults.

Lower vitamin D levels and VDR gene polymorphisms are also associated with susceptibility to obesity. As a molecular mechanism, vitamin D binds to the VDR protein, acting as a regulator in adipocyte differentiation and metabolism. Numerous single nucleotide polymorphisms (SNPs) exist within the VDR gene, altering the binding pattern of VDR to vitamin D or its analogs and related signaling pathways, thereby affecting adipocyte differentiation and metabolism (13). Previous studies based on NHANES data have revealed associations between vitamin D and sleep deprivation in diverse populations: a Mendelian randomization study indicated that lower vitamin D concentrations in the population may be associated with an increased risk of sleep disorders (14); another study explored the relationship between sleep patterns, vitamin D, and coronary heart disease, finding that serum 25-hydroxyvitamin D concentrations were inversely correlated with coronary heart disease risk, and this association was more significant and stable in participants with poor sleep patterns (*P*-interaction < 0.01) (15). While Danelly et al. (16) study broke through the limitations of direct associations between serum vitamin D and sleep duration by incorporating metal exposure interactions, it was constrained by a small study time span and limited population coverage, and failed to deeply explore potential mediating relationships. Notably, prior research has primarily focused on direct associations between vitamin D levels and sleep duration, as well as between sleep duration and obesity, leaving the underlying mediating mechanisms in these relationships largely unexplored. Our study systematically dissects these mechanisms for the first time, providing an innovative contribution to the field (17–19). In this context, we conducted a cross-sectional study involving 20,865 adult participants from the NHANES database between 2007 and 2018. Our aim was to investigate the mediating effect of BMI in vitamin D-related sleep deprivation, further exploring their combined impact on sleep deprivation. We hope this study will provide valuable insights for the prevention and treatment strategies of such conditions.

## Methods

### Data sources and study population

The National Health and Nutrition Examination Survey (NHANES, https://wwwn.cdc.gov/nchs/nhanes/search/default.aspx) is a nationally representative study that uses a complex, multistage probability sampling design to select a representative sample of the non-institutionalized civilian population in the United States. This survey aims to assess the health and nutritional status of Americans. NHANES is conducted by the National Center for Health Statistics (NCHS) of the Centers for Disease Control and Prevention (CDC) and is approved by the NCHS Institutional Review Board. All participants provided written informed consent.

**Figure 1:**
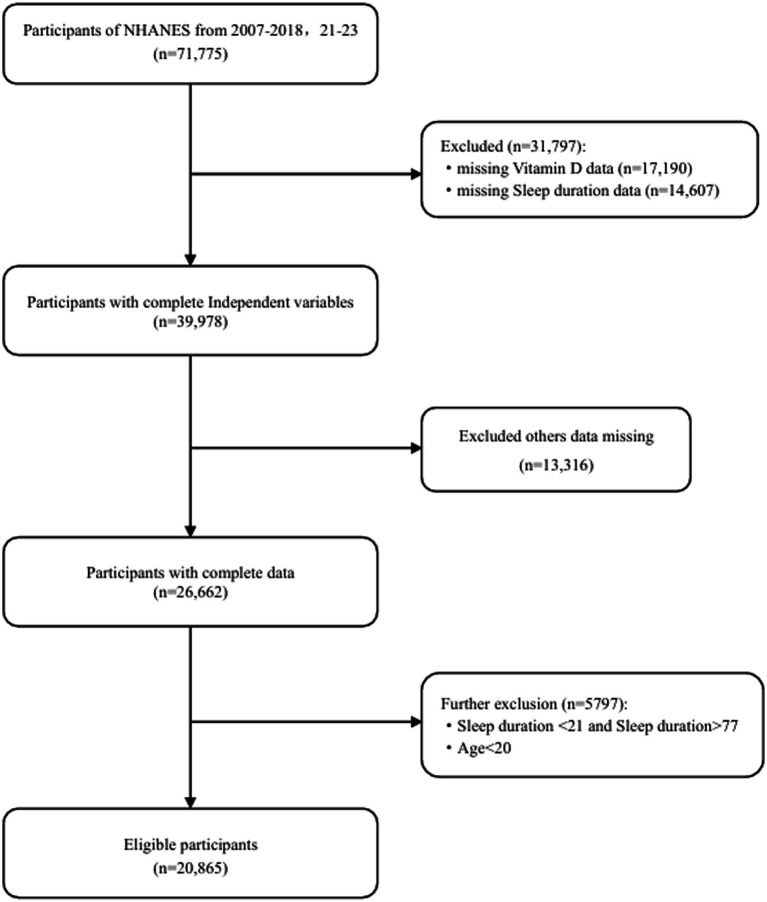
Flowchart of the participants included.

This study included data from six cycles of the NHANES database spanning 2007–2018,2021–2023 initially recruiting 71,775 adult participants. Due to the COVID-19 pandemic, the NHANES program suspended on-site operations in March 2020. Consequently, the data collection for the NHANES 2019–2020 cycle remained uncompleted. The collected data lacked national representativeness, and 100% of the participants had missing data on sleep duration, vitamin D, and BMI. Therefore, we excluded all data from the 2019–2020 period. Firstly, we excluded 31,797 participants due to missing data on vitamin D levels, sleep duration, or extreme data points. Subsequently, 13,316 participants with missing data on other covariates were also excluded. Finally, we excluded 5,797 participants whose sleep duration was identified as an outlier. Consequently, a total of 20,865 participants were included in this study (Figure 1).

### Determination of serum vitamin D concentration

The CDC method utilizes high-performance liquid chromatography–tandem mass spectrometry (HPLC-MS/MS) to quantitatively detect 25-hydroxyvitamin D3 (25OHD3), epi-25-hydroxyvitamin D3 (epi-25OHD3), and 25-hydroxyvitamin D2 (25OHD2) in human serum. This method is considered the gold standard for assessing vitamin D status. Chromatographic separation of these analytes is typically performed on one of three pentafluorophenyl (PFP) columns. The composition of the optimized chromatographic mobile phase varies slightly among the three columns but generally consists of 69 to 72% methanol aqueous solution. The composition of the solution added to the serum prior to extraction, the reconstitution solution, and the needle wash solution should match that of the mobile phase. For detailed experimental procedures, please refer to the 2022 laboratory methods (https://wwwn.cdc.gov/nchs/data/nhanes/2017-2018/labmethods/VID-J-MET-508.pdf).

Therefore, in our study, “vitamin D” refers to serum vitamin D, specifically the concentration of 25OHD2 and 25OHD3 (nmol/L) in the blood. These represent the primary metabolites of vitamin D, namely 25-hydroxyvitamin D2 and 25-hydroxyvitamin D3.

### Sleep duration measurement

Sleep duration was determined by a self-reported item: “How long do you usually sleep at night?” This question was administered by trained interviewers using a Computer-Assisted Personal Interview (CAPI) system at participants’ homes. The CAPI system included limited built-in consistency checks during programming to reduce data entry errors. As described in “Data Processing and Editing,” additional edits were made after data collection to ensure the integrity, consistency, and usability of the data for analysis.

Sleep duration was categorized into two groups: sleep deprivation and adequate sleep duration, with sleep deprivation defined as less than 7 h per night.

### Assessment of BMI

Anthropometric data were collected by trained health technicians at the Mobile Examination Center (MEC). Examiners first measured the participants’ height and weight, and then calculated the precise BMI using the formula BMI = weight (kg) / height (m)^2.

### Assessment of covariations

The covariates in this study included: age, gender, race, education level, family income-to-poverty ratio, HDL, smoking, alcohol consumption, diabetes, systolic blood pressure, congestive heart failure, coronary heart disease, and cancer. Among these, age, systolic blood pressure, and HDL are continuous variables, while the rest are categorical variables.

### Statistical analysis

In our study, since the analysis incorporated both laboratory measurements and questionnaire data, the Full Sample 2 Year MEC Exam Weight (WTMEC2YR) was used for weighting. Given that data from seven two-year cycles were utilized, we divided the WTMEC2YR weight by 7 to adjust for the combined sample size. Weighted regression analysis was conducted using the survey package to ensure the generalizability of the results and to minimize bias arising from the complex sampling design.

In the baseline demographic table, continuous variables are presented as means ± standard deviations, and categorical variables are shown as counts (percentages), with assessments of significant differences between groups. The associations between vitamin D, BMI, and sleep deprivation were evaluated using logistic regression models, specifically through three models: Model 1: Unadjusted for confounders; Model 2: Adjusted for age, gender, and race; Model 3: Adjusted for age, gender, race, education level, family income-to-poverty ratio, HDL, smoking, alcohol consumption, diabetes, and cancer. Restricted cubic splines (RCS) were used to determine the risk associations of BMI and vitamin D levels with sleep duration. Mediation analysis was employed to assess the mediating role of BMI in vitamin D-related sleep deprivation. Subgroup analyses were conducted to explore the relationships between vitamin D and sleep deprivation across different subgroups.

All analyses were performed using R version 4.3.3. A *p*-value < 0.05 was considered statistically significant.

## Results

### Baseline characteristics

Table 1 presents the characteristics of participants grouped by whether their sleep duration was adequate (>7 h). The cohort included 49% male participants and 44% non-Hispanic White people. Among this group, 2,715 individuals (39%) exhibited inadequate sleep duration. When stratified by adequate versus inadequate sleep duration, differences were observed in all variables except for systolic blood pressure, coronary heart disease, and congestive heart failure (*p* < 0.05). Notably, significant differences (*p* < 0.001) were found between groups for covariates such as age, sex, PIR, race, education level, vitamin D levels, BMI, waist circumference, HDL, smoking, and alcohol consumption.

**Table 1:** Characteristics of the study population based on sleep duration groups in NHANES.

Characteristic	Sleep duration			*p*-value^2^
	Overall *N* = 20,865^1^	Insufficient *N* = 7,029^1^	Sufficient *N* = 13,836^1^	
Age, (years)	49 ± 18	49 ± 17	50 ± 18	<0.001
Sex, *n* (%)				<0.001
Male	10,181 (49%)	3,568 (51%)	6,613 (48%)	
Female	10,684 (51%)	3,461 (49%)	7,223 (52%)	
Race, *n* (%)				<0.001
Mexican American	3,231 (15%)	989 (14%)	2,242 (16%)	
Other Hispanic	2,258 (11%)	792 (11%)	1,466 (11%)	
Non-Hispanic White	9,119 (44%)	2,715 (39%)	6,404 (46%)	
Non-Hispanic Black	4,192 (20%)	1,873 (27%)	2,319 (17%)	
Other Race	2,065 (9.9%)	660 (9.4%)	1,405 (10%)	
Ratio of family in come to poverty, *n* (%)				<0.001
From 0 to 1	4,947 (24%)	1,787 (25%)	3,160 (23%)	
From 1 to 3	10,706 (51%)	3,566 (51%)	7,140 (52%)	
Over3	5,212 (25%)	1,676 (24%)	3,536 (26%)	
Education level, *n* (%)				<0.001
Less than 9th grade	2,064 (9.9%)	674 (9.6%)	1,390 (10%)	
9-11th grade	3,098 (15%)	1,147 (16%)	1,951 (14%)	
High school graduate/GED or equivalent	5,163 (25%)	1,802 (26%)	3,361 (24%)	
Some college or AA degree	6,681 (32%)	2,335 (33%)	4,346 (31%)	
College graduate or above	3,859 (18%)	1,071 (15%)	2,788 (20%)	
Smoke, *n* (%)	9,833 (47%)	3,546 (50%)	6,287 (45%)	<0.001
Drink, *n* (%)	13,756 (66%)	4,777 (68%)	8,979 (65%)	<0.001
Diabetes, *n* (%)	2,811 (13%)	1,010 (14%)	1,801 (13%)	0.007
Congestive heart failure, *n* (%)	685 (3.3%)	248 (3.5%)	437 (3.2%)	0.2
Coronary heart disease, *n* (%)	867 (4.2%)	280 (4.0%)	587 (4.2%)	0.4
Cancer, *n* (%)	2,031 (9.7%)	619 (8.8%)	1,412 (10%)	0.001
Vitamin D, *n* (%)				<0.001
<50	6,570 (31%)	2,595 (37%)	3,975 (29%)	
50-72	6,797 (33%)	2,294 (33%)	4,503 (33%)	
≥72	7,498 (36%)	2,140 (30%)	5,358 (39%)	
BMI (kg/m^2^)	29 ± 7	30 ± 7	29 ± 7	<0.001
Waistline (cm)	100 ± 17	101 ± 17	100 ± 16	<0.001
Systolic blood pressure (mmHg)	125 ± 19	125 ± 18	125 ± 19	0.4
HDL (mmol/L)	1.35 ± 0.41	1.34 ± 0.41	1.36 ± 0.41	<0.001

^1^Mean ± SD; *n* (%).

^2^Wilcoxon rank sum test; Pearson's Chi-squared test.

### Assessment of vitamin D, BMI levels, and sleep deprivation risk

Through the patient baseline characteristics table, we identified that the indicators age, gender, race, education level, family, BMI, income-to-poverty ratio, HDL, smoking, alcohol consumption, diabetes, and cancer exhibited differences across various groups. Initially, we conducted univariate regression analyses for each factor to determine whether these variables were correlated with and significantly associated with sleep deprivation. The results showed that all variables had significant associations with sleep deprivation (Table 1). This indicates that these variables need to be included in further model analyses for multivariate regression analysis. Additionally, we used the Variance Inflation Factor (VIF) to assess collinearity among the variables in the multivariate analysis (Table 1 and Supplementary Figure S1). The results revealed that the VIF values ranged from 1.07 to 1.32, suggesting weak collinearity among the variables, which enhances the credibility of the model’s effects.

In Table 2, after adjusting for confounding factors and taking sufficient vitamin D levels as the reference, participants with vitamin D deficiency had a higher risk of sleep deprivation: Model 1 (OR = 1.44; 95% CI: 1.36–1.54; *p* < 0.001), Model 2 (OR = 1.30; 95% CI: 1.22–1.39; *p* < 0.001), Model 3 (OR = 1.26; 95% CI: 1.18–1.35; *p* < 0.001). Taking BMI < 75th percentile as the reference, participants with BMI > 75th percentile showed a higher risk in Model 1 (OR = 1.21; 95% CI: 1.13–1.29; *p* < 0.001), Model 2 (OR = 1.18; 95% CI: 1.11–1.27; *p* < 0.001), and Model 3 (OR = 1.14; 95% CI: 1.06–1.22; *p* < 0.001). Combining vitamin D status with BMI, taking vitamin D deficiency and BMI > 75th percentile (<33.1 kg/m^2^) as the reference, participants with adequate vitamin D and BMI < 75th percentile exhibited the lowest risk of sleep deprivation (OR = 0.7, 95% CI: 0.64–0.77; *p* < 0.001). These results suggest that under various adjustment conditions, the risk of sleep deprivation is significantly reduced to 0.7 times when vitamin D levels are elevated and BMI is < 75th percentile.

**Table 2:** ORs (95% CIs) for sleep disorders according to the Vitamin D and BMI.

Characteristic	Model 1			Model 2			Model 3		
	OR^1^	95% CI^1^	*p*-value	OR^1^	95% CI^1^	*p*-value	OR^1^	95% CI^1^	*p*-value
Vitamin D									
Sufficient	—	—		—	—		—	—	
Inadequate	1.44	1.36, 1.54	<0.001	1.30	1.22, 1.39	<0.001	1.26	1.18, 1.35	<0.001
BMI									
<75th percentile	—	—	—	—	—	—	—	—	—
>75th percentile	1.21	1.13, 1.29	<0.001	1.18	1.11, 1.27	<0.001	1.14	1.06, 1.22	<0.001
Group									
VD Inadequate & BMI>75%	—	—	—	—	—	—	—	—	—
VD Sufficient & BMI>75%	0.65	0.57, 0.74	<0.001	0.72	0.63, 0.82	<0.001	0.72	0.63, 0.83	<0.001
VD Inadequate & BMI<75%	0.83	0.77, 0.90	<0.001	0.83	0.77, 0.90	<0.001	0.86	0.79, 0.93	<0.001
VD Sufficient & BMI<75%	0.59	0.54, 0.65	<0.001	0.66	0.60, 0.72	<0.001	0.70	0.64, 0.77	<0.001

^1^OR = Odds Ratio, CI = Confidence Interval.

Model 1: Unadjusted.

Model 2: Adjusted for age, sex, and race.

Model 3: Adjusted for age, sex, race, education, PIR, smoking status, drink, HDL, diabetes, cancer, congestive heart failure, coronary heart disease, sleep duration.

**Figure 2:**
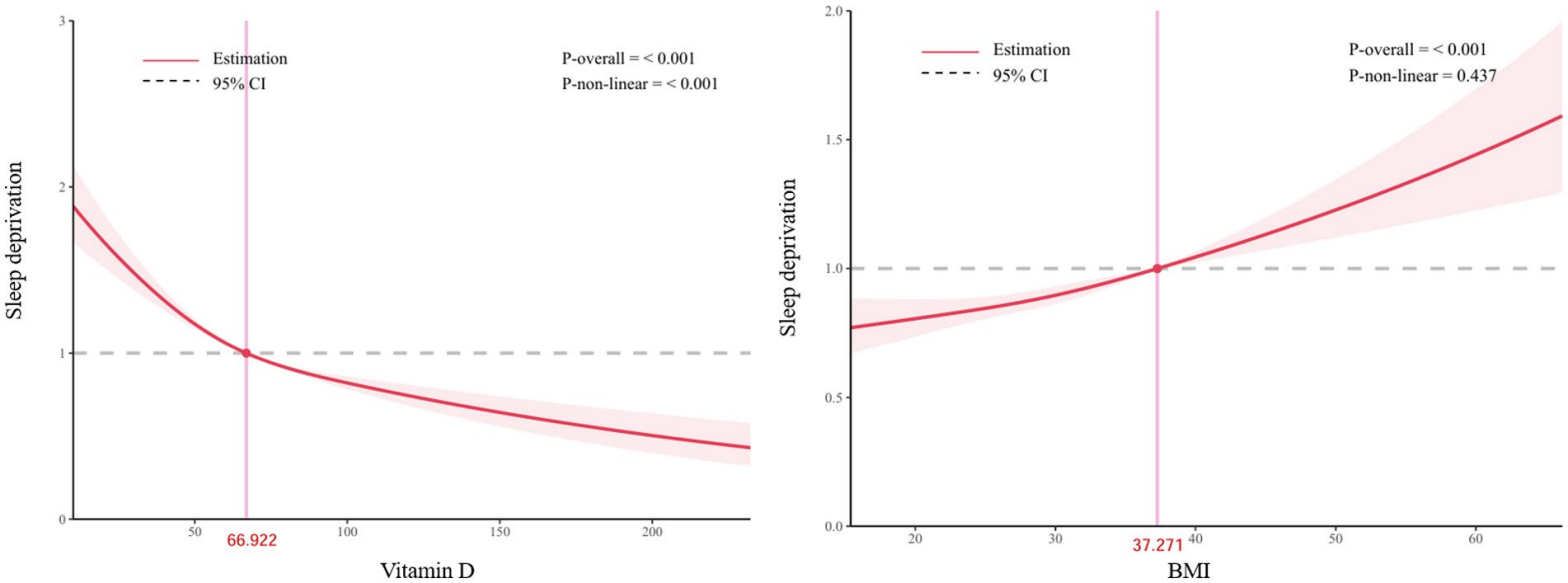
RCS model between BMI, vitamin D and sleep deprivation.

Figure 2 shows that in the RCS analysis, there is a significant nonlinear relationship between vitamin D and the risk of sleep deprivation (*p* < 0.001). The relationship between BMI and the risk of sleep deprivation can be approximated as linear (*P*-overall≤0.001, *P*-non-linear = 0.437).

### Assessment of mediation effect

**Figure 3:**
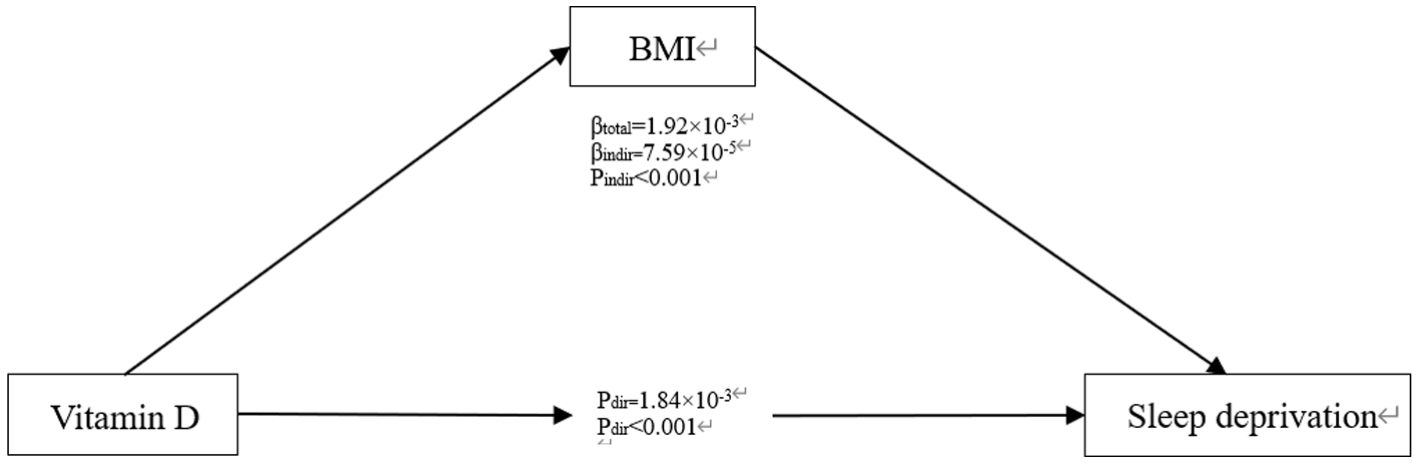
Mediating role of BMI in serum vitamin D related sleep deprivation.

Finally, to explore the potential mediating role of vitamin D and BMI, a mediation analysis was conducted (Figure 3). The results indicate that vitamin D has a significant direct effect on sleep deprivation (*β* total = 1.92*10^−3^, *P* dir < 0.001), consistent with our previous findings from logistic regression. The influence of vitamin D on the risk of sleep deprivation through BMI is also significant (*β* indir = 7.59*10^−5^, *P* indir < 0.001), suggesting that vitamin D may affect the risk of sleep deprivation both directly and indirectly through its impact on BMI.

### Subgroup analysis of the effect of serum vitamin D on sleep deprivation

**Figure 4:**
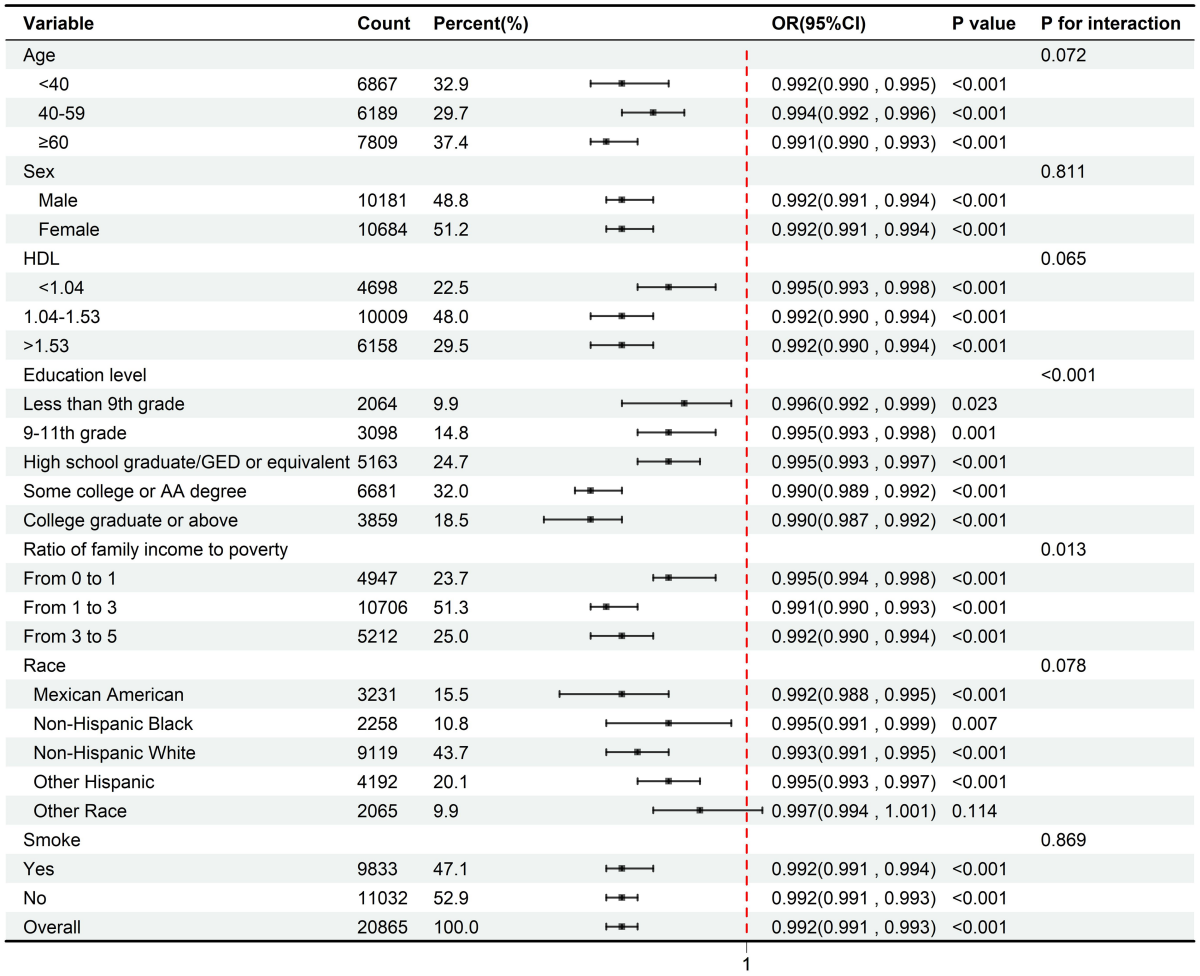
Subgroup analysis of the effect of serum vitamin D on sleep deprivation.

Figure 4 illustrates the association between serum vitamin D and the risk of sleep deprivation through separate logistic regression analyses stratified by age, gender, race, education level, the ratio of family income to poverty, HDL, and smoking status.

In Figure 4, the interaction analysis shows that there are statistically significant differences among the subgroups stratified by educational level (*P* for interaction<0.001) and the ratio of family income to poverty (*P* for interaction = 0.013), while other subgroup differences are not statistically significant. The reduction in the risk of sleep deprivation associated with increased vitamin D levels is more pronounced among older adults (OR = 0.991, 95% CI = 0.99–0.993), Individuals with some college or AA degree (OR = 0.99, 95% CI = 0.989–0.992) or college graduate or above qualifications (OR = 0.99, 95% CI = 0.987–0.992), and Mexican Americans (OR = 0.992, 95% CI = 0.988–0.995). Despite these observations, the relationship does not significantly differ across subgroups (*p* > 0.05).

## Discussion

This cross-sectional analysis based on NHANES (2007–2018, 21–23, *n* = 20,865) investigated the complex relationships among 25(OH)D status, obesity, and sleep deprivation in U. S. adults. The main findings revealed that, even after adjusting for sociodemographic, lifestyle, and clinical confounders, vitamin D insufficiency and elevated BMI were independently and synergistically associated with the prevalence of sleep deprivation. Compared with the insufficient state, the combined protective effect of optimal 25(OH)D (nmol/L) and BMI < 75th percentile (<33.1 kg/m^2^) reduced the risk of sleep deprivation (OR = 0.7, 95% CI: 0.64–0.77). Subgroup analyses further highlighted that the protective association between higher vitamin D and reduced sleep deprivation risk was most pronounced inolder adults, individuals with higher education, and Mexican Americans. These results underscore the importance of addressing both metabolic (BMI) and nutritional (vitamin D) factors in sleep health interventions.

The mechanisms by which vitamin D influences sleep have been previously suggested to involve the presence of 1α-hydroxylase and 1,25-dihydroxyvitamin D receptors (VDR) in the brain. Studies have shown that 1,25-dihydroxyvitamin D can regulate the expression of circadian clock genes (e.g., BMAL1 and PER2) in adipose-derived stem cells (12, 20). These vitamin D receptors are highly expressed in areas such as the anterior and posterior hypothalamus, substantia nigra, midbrain periaqueductal gray, raphe nuclei, and the pontine and caudal reticular nuclei, which appear to coordinate sleep–wake states and muscle atonia during sleep. Vitamin D can bind to these receptors, thereby modulating sleep and affecting sleep quality. Another pathway by which vitamin D may influence sleep quality is through its effect on tryptophan hydroxylase (TPH)-2, which regulates the conversion of tryptophan to 5-hydroxytryptophan (21).

Mechanistic studies demonstrate that 25-hydroxyvitamin D (25(OH)D) modulates obesity through distinct pathways: First, it binds to the vitamin D receptor (VDR) to upregulate lipolytic genes while suppressing adipogenic gene expression, thereby inhibiting adipocyte differentiation and lipid accumulation (22); second, it rapidly elevates intracellular calcium levels to activate calcium-mediated signaling cascades, triggering apoptosis in mature adipocytes (23). Furthermore, 25(OH)D enhances energy expenditure and mitochondrial function in adipocytes by potentiating NAD-SIRT1 pathway activity (24).

Sleep deprivation include obstructive sleep apnea (OSA) and obesity hypoventilation syndrome (OHS), with obesity being the primary risk factor for sleep-disordered breathing (25). Mechanistically, excessive fat accumulation in the neck and reduced lung volumes significantly increase the risk of upper airway obstruction, leading to intermittent nocturnal hypoxia and hypercapnia during sleep. Additionally, impaired respiratory muscle function and blunted respiratory drive further exacerbate ventilatory dysfunction, ultimately resulting in sustained daytime hypercapnia during wakefulness (26, 27). These pathophysiological changes interact, forming a vicious cycle that disrupts sleep and clinically manifests as OSA and OHS.

However, there is currently a lack of large-scale population-based studies on the relationship between serum vitamin D and sleep duration in adults. Previous research, such as that by Kumars Pourrostami et al. on Iranian children, supports our findings of a significant association between vitamin D and sleep (28) Yuning Xie et al. (18) demonstrated that adequate VD3 levels combined with good sleep and normal sleep duration (7–8 h) could enhance cognitive function in elderly Chinese individuals; however, they did not further explore the relationship of BMI in VD levels associated sleep duration, which our study addresses.

There are limitations to our study: (1) Cross-Sectional Design: The study’s cross-sectional nature limits the ability to infer causality. While associations between vitamin D levels, BMI, and sleep deprivation were observed, it remains unclear whether vitamin D deficiency causes sleep disturbances or if sleep disturbances contribute to lower vitamin D levels. Longitudinal studies are needed to better understand the directionality of these relationships; (2) Self-Reported Sleep Duration: Sleep duration was determined through a self-reported questionnaire, which is subject to recall bias and inaccuracies. Objective measures of sleep, such as polysomnography or actigraphy, could provide more accurate assessments of sleep duration and quality, enhancing the validity of the results. (3) Lack of Specific Mechanistic Data: Although the study explored potential mediation by BMI, the precise biological mechanisms by which vitamin D influences sleep remain unclear. Further research at the molecular level, including studies on circadian rhythm regulation and the role of vitamin D receptors in the brain, is needed to elucidate the underlying mechanisms.

However, we believe this study makes several important contributions to the existing literature. Firstly, while previous research has explored the relationship between vitamin D and sleep, most studies have not adequately considered BMI as a potential mediator. By revealing the mediating role of BMI in the relationship between vitamin D and sleep deprivation, we provide further insight into the complex interactions between these factors and offer new directions for future research. Secondly, our study demonstrates that the combined effect of adequate vitamin D levels and a lower BMI significantly reduces the risk of sleep deprivation. This finding offers a new perspective for public health interventions. Improving vitamin D levels and controlling body weight may help reduce the incidence of sleep problems, particularly among males, older adults, and individuals with higher education levels. These results provide practical implications for public health policies and individualized health interventions.

## Conclusion

In conclusion, our research findings strongly suggest a significant association between serum vitamin D levels in adult Americans and sleep deprivation (*p* < 0.001). Moreover, this study has successfully elucidated the mediating and regulatory mechanisms underlying the relationship between serum vitamin D and sleep deprivation in adults. Specifically, serum vitamin D may indirectly impact the occurrence of sleep deprivation by influencing body - mass index (BMI). These findings offer novel perspectives on the relationship between vitamin D and sleep, underscoring BMI as a modifiable factor in the causal chain. This connection can be exploited for targeted interventions.

## Data Availability

Publicly available datasets were analyzed in this study. This data can be found at: https://wwwn.cdc.gov/nchs/nhanes/default.aspx, NHANES.
