# Supplementary figures and images for: The mediating effect of BMI in serum vitamin D related sleep deprivation based on the NHANES database
Source: Front Nutr. 2025 Jun 10;12:1571985. doi: 10.3389/fnut.2025.1571985 (PMC12185422; doi:10.3389/fnut.2025.1571985)

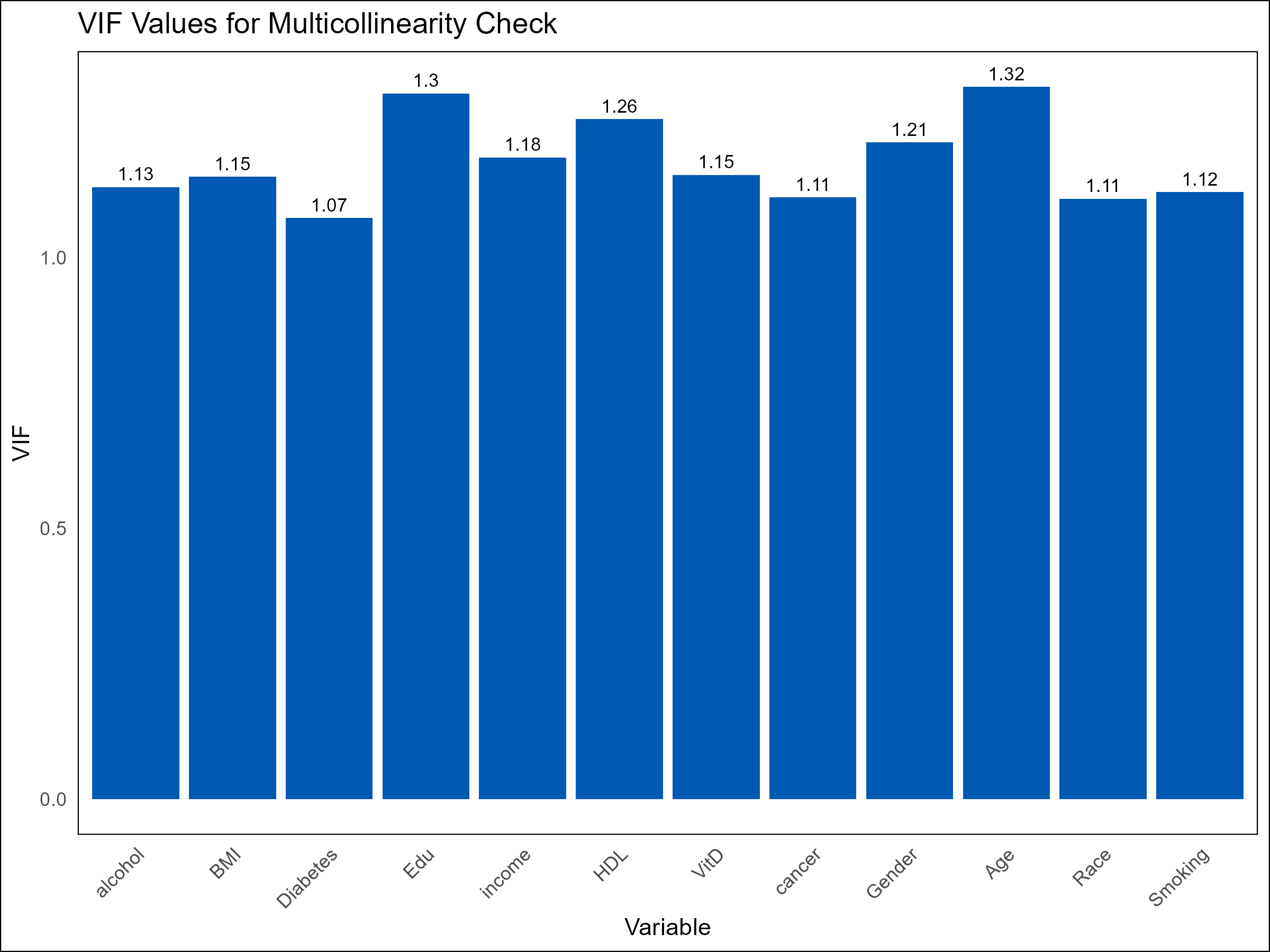

Supplement: Supplementary file 1 [file Image_1.png]
